# Supplementary material for: The AAA+ ATPase RavA and its binding partner ViaA modulate E. coli aminoglycoside sensitivity through interaction with the inner membrane
Source: Nat Commun. 2022 Sep 20;13:5502. doi: 10.1038/s41467-022-32992-9 (PMC9489729; doi:10.1038/s41467-022-32992-9)
Supplement: Supplementary file 1 — Supplementary Information [file 41467_2022_32992_MOESM1_ESM.pdf]

Supplementary Figure and Legends

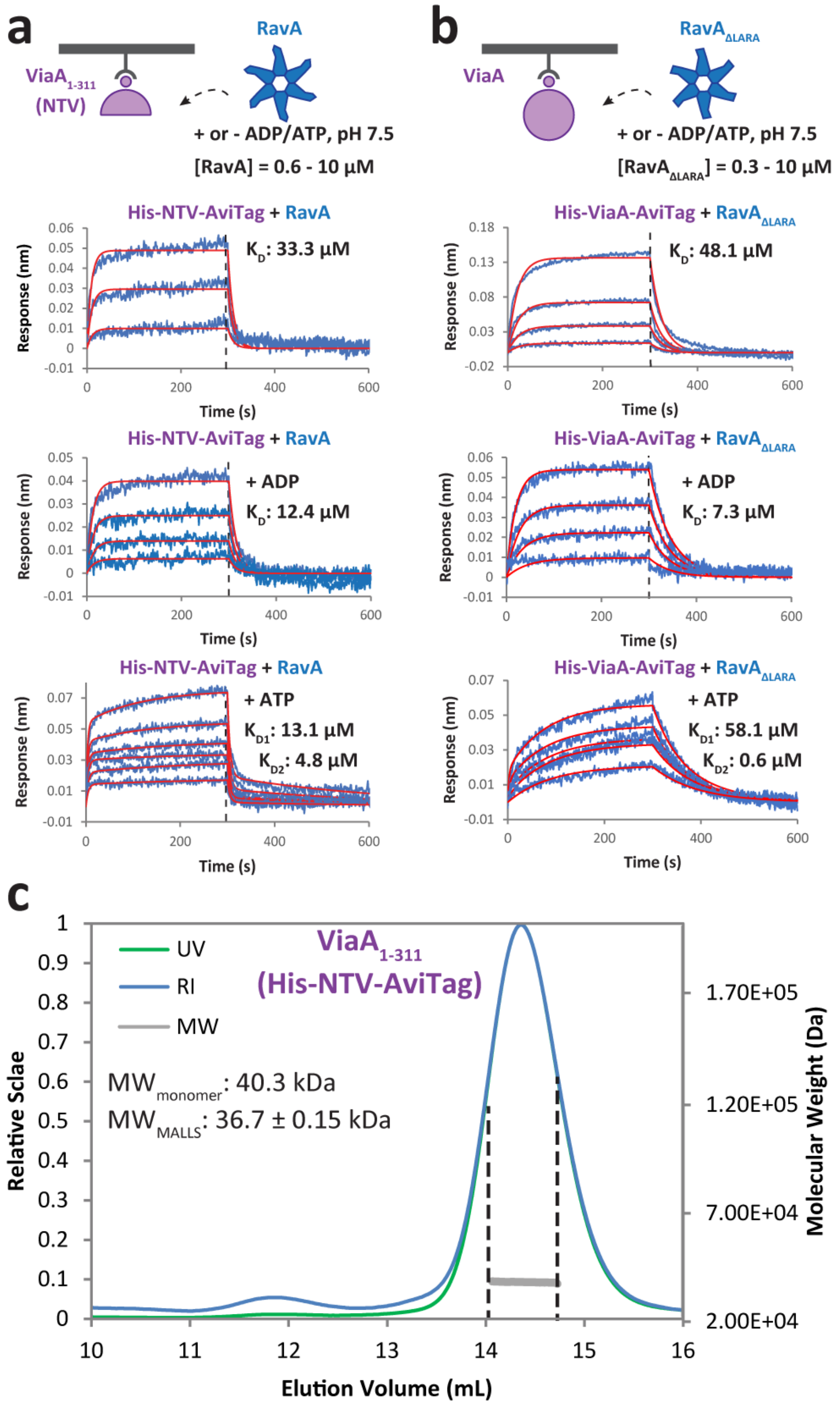

Supplementary Figure 1: Comparison between the N-terminal domain of ViaA (NTV) and full-length ViaA in terms of interaction with RavA and oligomeric state. A) BLI measurements of His-NTV-AviTag coupled on BLI biosensors and RavA, with or without added ADP/ATP. B) BLI measurements of AviTag-ViaA-His coupled on BLI biosensors and $RavA_{\Delta LARA}$, with or without added ADP/ATP. For A) and B), the blue curves correspond to the measured signal while the red curves correspond to the calculated fit using a 1:1 (no nucleotide, ADP) or 2:1 heterogeneous ligand binding (ATP) interaction model. BLI experiments were performed in triplicate, and one representative experiment is shown in each case. C) Molecular mass determination of His-NTV-AviTag by SEC-MALLS. The differential refractive index (RI) signal is plotted (left axis, blue curve) along with the UV signal (UV, left axis, green curve) and the determined molecular weight (MW, right axis, grey curve). The theoretical monomer MW and the MW as determined by MALLS are annotated on the left-hand side of the plot. The MALLS experiment was performed once. Source data for Supplementary Figure 1 are provided as a Source Data file.

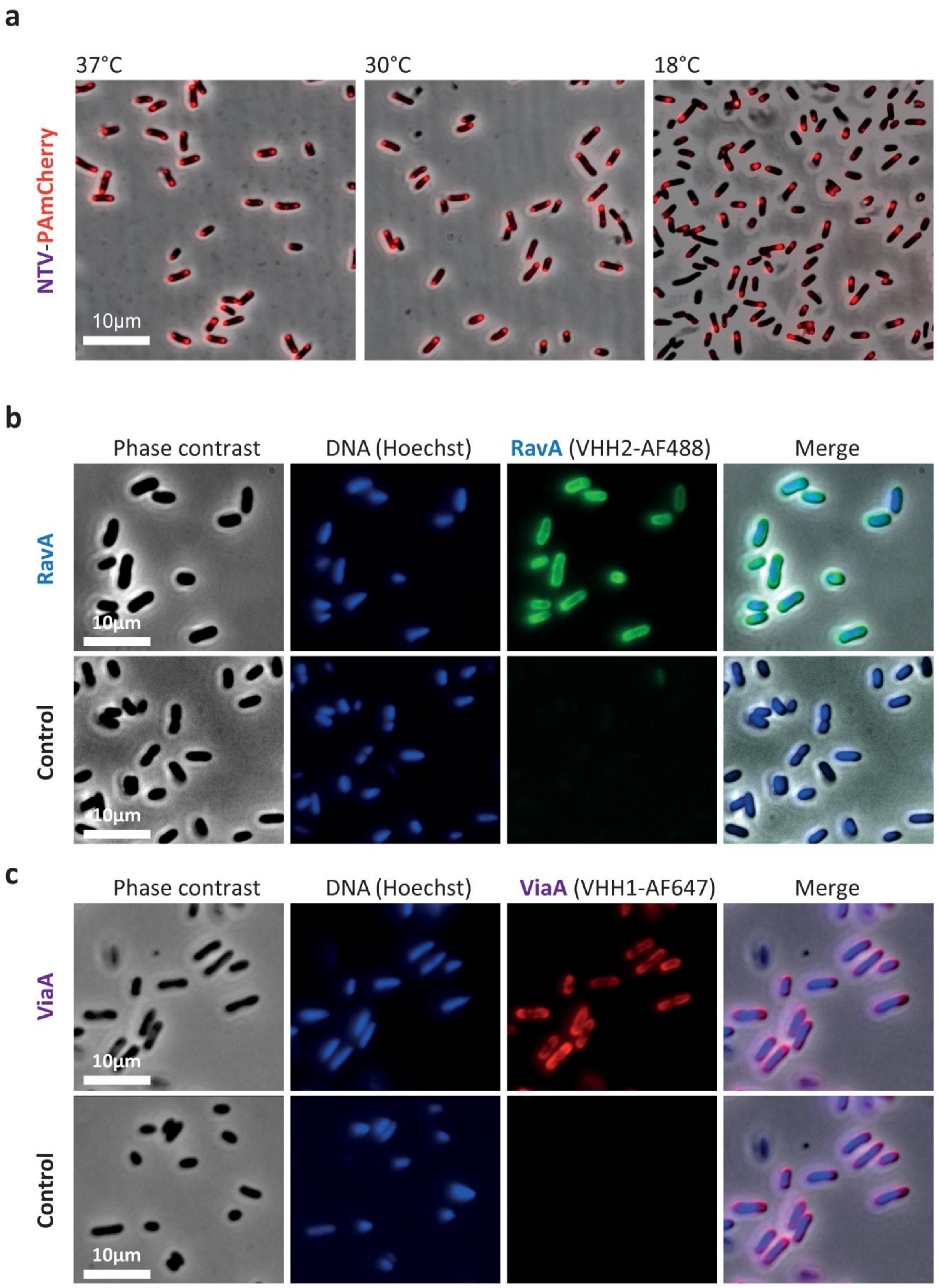

Supplementary Figure 2: Additional optical imaging studies. A) Single molecule localisation microscopy imaging of *E. coli* cells overexpressing the N-terminal domain of ViaA (NTV) fused to PAmCherry by PALM at 37° C (left), 30° C (middle) and 18° C (right). Each experiment was performed in triplicate, and one representative experiment is shown. B & C) Wide field imaging of *E. coli* cells overexpressing RavA (B) or ViaA (C) using phase contrast, DNA (Hoechst) staining or anti-RavA (VHH2-AF488) or anti-ViaA (VHH1-AF647) nanobodies coupled to Alexa Fluor dyes. Each experiment was performed in triplicate, and one representative experiment is shown.

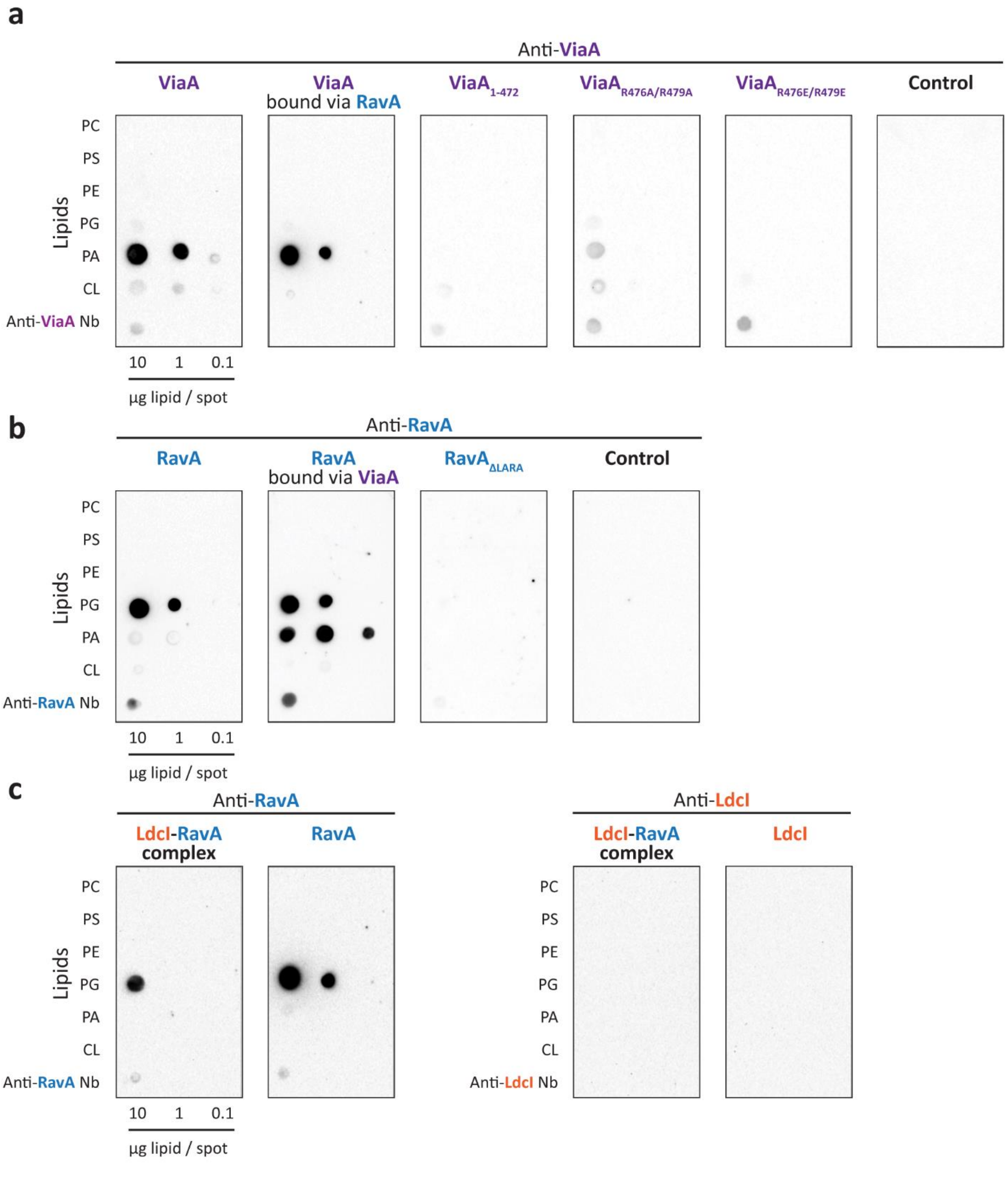

Supplementary Figure 3: Extended dot-blot assays. Dot-blot assays using purified ViaA constructs (A: ViaA, ViaA$_{1\text{-}472}$, ViaA$_{R476E/R479E}$), purified RavA constructs (B: RavA, RavA$_{\Delta LARA}$), or purified LdcI-RavA complex (C) visualized using anti-ViaA (A) and anti-RavA (B & C) or anti-LdcI (C) antibodies and a secondary HRP-antibody conjugate (PC: phosphatidylcholine, PS: phosphatidylserine, PE: phosphatidyl-ethanolamine, PG: phosphatidylglycerol, PA: phosphatidic acid, CL: cardiolipin).

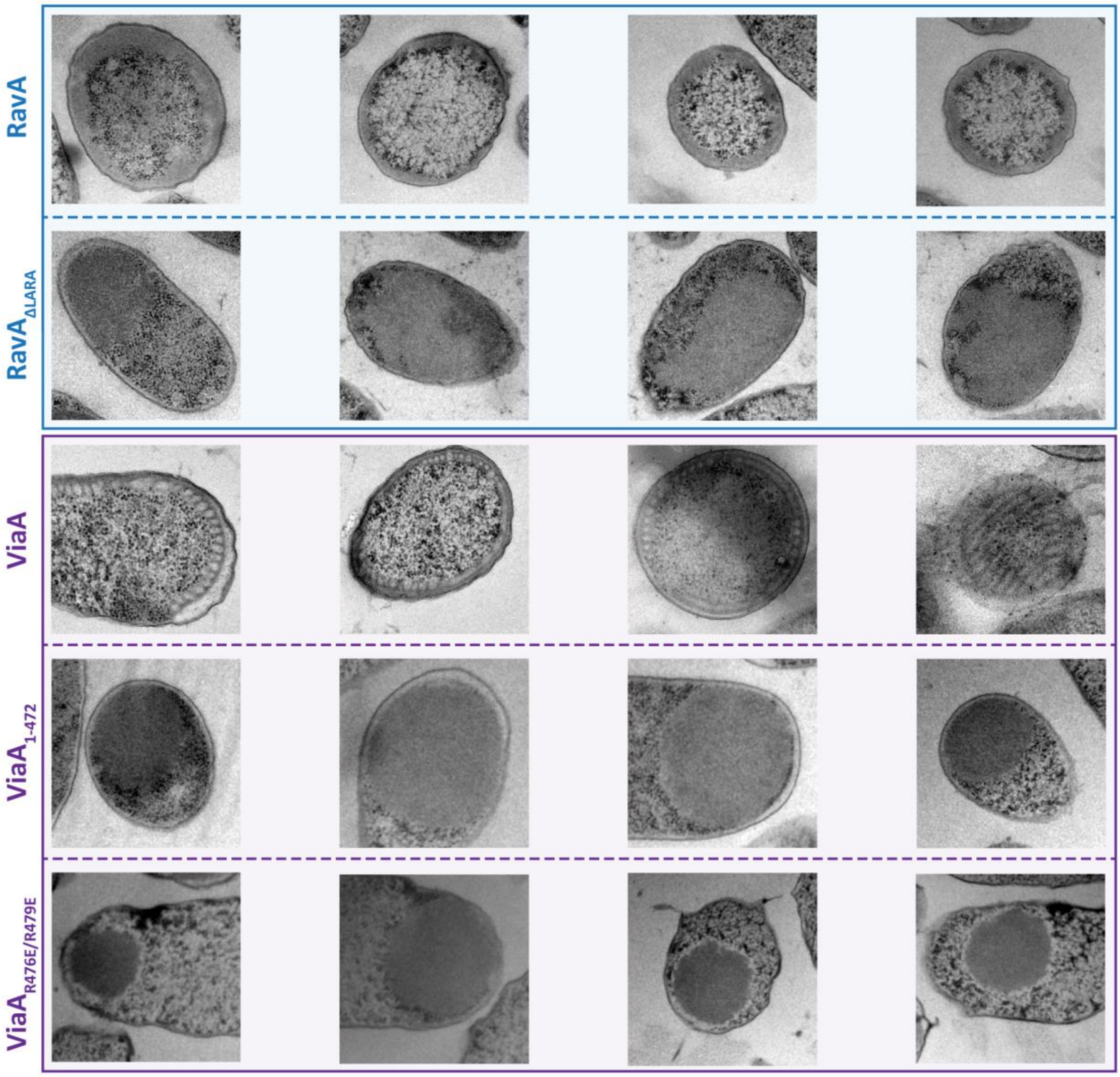

Supplementary Figure 4: Additional TEM imaging studies. TEM imaging of high pressure frozen, freeze-substituted and sectioned *E. coli* cells overexpressing different RavA (blue square: RavA, RavA$_{\Delta LARA}$) or ViaA (purple square: ViaA, ViaA$_{1-472}$ or ViaA$_{R476E/R479E}$) constructs. Each experiment was performed in triplicate, and one representative experiment is shown.

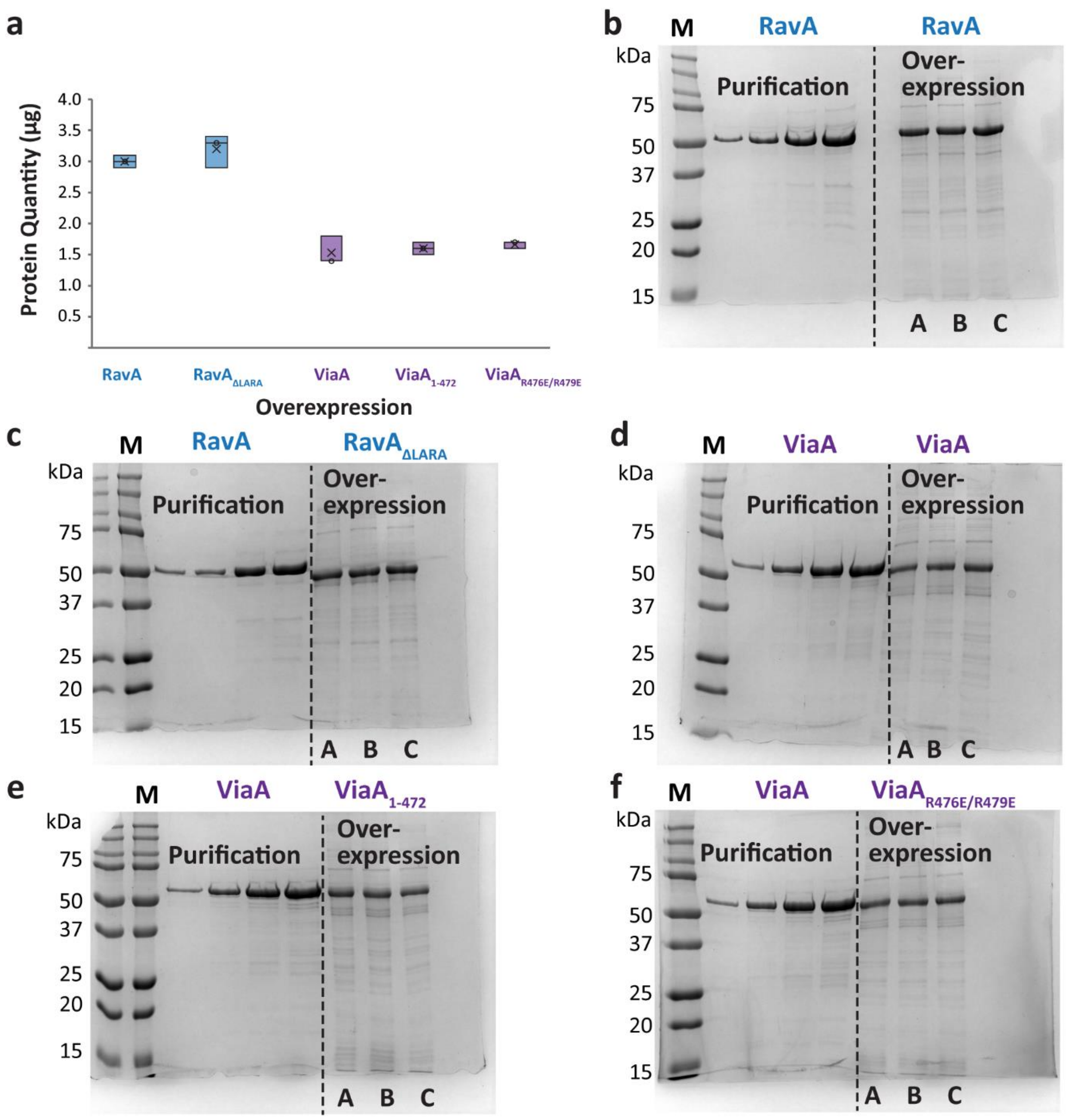

Supplementary Figure 5: Overexpression level of the different constructs used for the TEM imaging in Supplementary Figure 4 and for the lipid quantification in Supplementary Figure 6. A) Box & Whisker chart showing the determination of protein quantity of overexpressed RavA, $RavA_{\Delta LARA}$, ViaA, $ViaA_{1\text{-}472}$ and $ViaA_{R476E/R479E}$, based on densitometric analysis of overexpression bands (three biological repeats) on SDS-PAGE gels shown in B (RavA), C ($RavA_{\Delta LARA}$), D (ViaA), E ($ViaA_{1\text{-}472}$) and F ($ViaA_{R476E/R479E}$), using Image Lab software. The Box & Whisker chart in (A) shows all points (n=3) within a box indicating the mean (cross), median, 25th and 75th percentiles and whiskers down to the minimum and up to the maximum value. Source data for Supplementary Figure 5 are provided as a Source Data file.

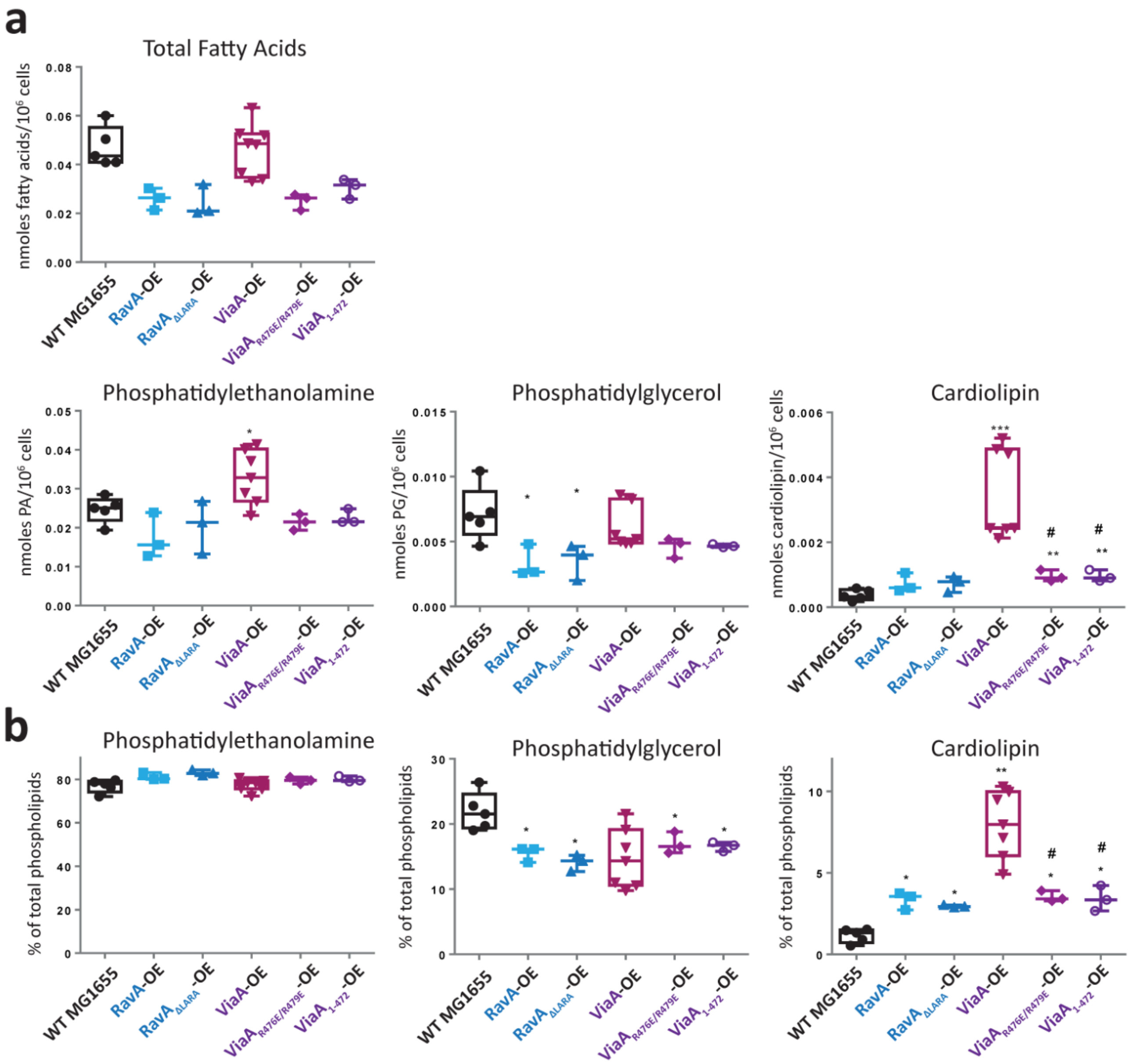

Supplementary Figure 6: Quantification of phospholipids in different RavA and ViaA overexpressing strains. Quantification of fatty acid levels (total, phosphatidylethanolamine, phosphatidylglycerol or cardiolipin) in nmol of fatty acid per million cells (A) or in percent of total phospholipid fatty acid (B) by TLC and GC-FID/MS in wild-type (WT) MG1655 *E. coli* cells and MG1655 *E. coli* cells overexpressing RavA, $RavA_{\Delta LARA}$, ViaA, $ViaA_{R476E/R479E}$ and $ViaA_{1\text{-}472}$, visualized by Tukey representations, showing all points with a box indicating the median, 25th and 75th percentiles and whiskers down to the minimum and up to the maximum value. Each point represents a biological repeat where n=3 for all lines except for WT where n=5 and for ViaA-OE (AviTag-ViaA-His) where n=7 for phosphatidylethanolamine, phosphatidylglycerol and cardiolipin and n=8 for total fatty acids. The overexpressing (OE) series were compared with the control WT MG1655 series using an unpaired two-sided nonparametric Mann-Whitney test. Significant difference with the control is shown by * for p value < 0.05 (p value = 0.0357 for total fatty acids, for phosphatidylethathanolamine p value = 0.048, for phosphatidylglycerol p value = 0.037, for cardiolipin p value = 0.0357 for $ViaA_{1\text{-}472}$ and $ViaA_{R476E/R479E}$) or ** for p value < 0.01 (p value = 0.0025. The # indicates a significant difference (p value = 0.0167) with ViaA-OE. In B, the bottom left panel showing the percentage of CL is identical to the Figure 4B and is shown here again to facilitate visual comparison between the three phospholipids. Source data for Supplementary Figure 6 are provided as a Source Data file.

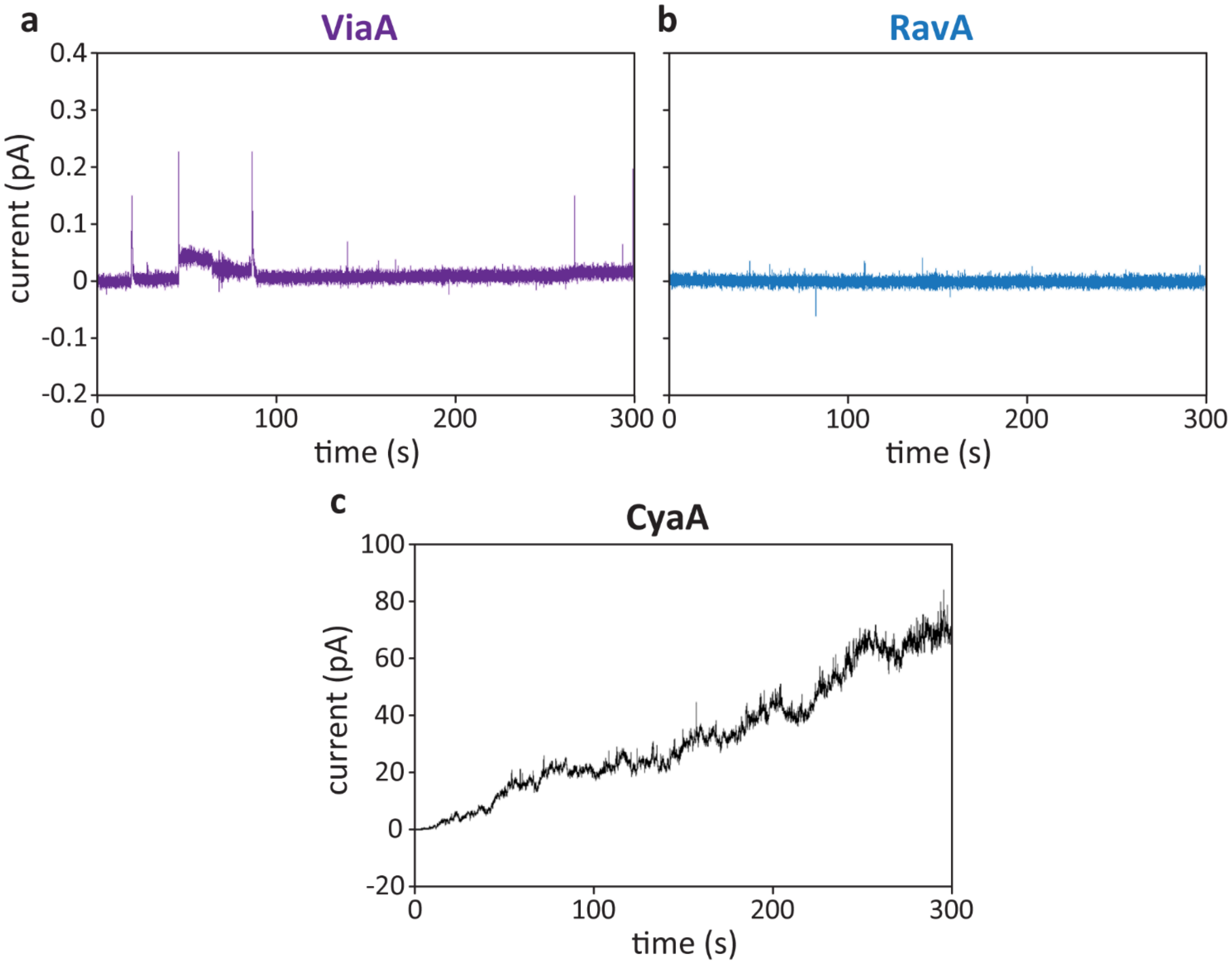

Supplementary Figure 7: Overall membrane activities of ViaA, RavA and the *B. pertussis* pore-forming CyaA toxin as measured by planar lipid bilayer technique. The ViaA (a) and RavA (b) proteins were diluted to a final concentration of 2 nM and exposed to a lipid membrane containing PA, PG and PE lipids in molar ratio of 10:45:45. The CyaA toxin (c), used as a positive control for membrane pore formation, was diluted to a final concentration of 250 pM and exposed to an asolectin membrane. The aqueous phase contained 10 mM Tris-HCl (pH 7.4), 150 mM KCl and 2 mM $CaCl_2$; the applied voltage was 50 mV; the temperature was 25 °C. The membrane current recordings were processed using a 10 Hz filter. Source data for Supplementary Figure 7 are provided as a Source Data file.

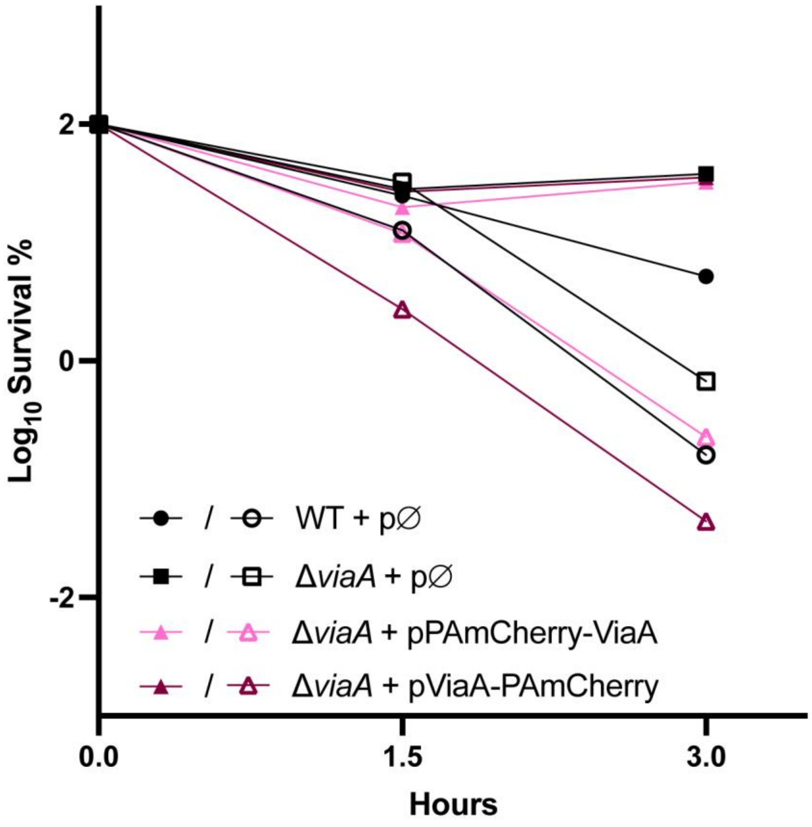

Supplementary Figure 8: Complementation of Δ*viaA* strain with plasmids expressing ViaA - PAmCherry or PAmCherry-ViaA renders the cells more sensitive to Gm. Survival of WT MG1655 (WT + empty vector p∅), Δ*viaA* + p∅, Δ*viaA* supplemented with PAmCherry-ViaA plasmid (Δ*viaA* + pPAmCherry-ViaA) or with ViaA-PAmCherry plasmid (Δ*viaA* + pViaA-PAmCherry), 1.5 and 3 hours after addition of 16 µg/mL Gm. Survival, measured by colony-forming units (CFU) per mL, was normalized relative to time zero at which Gm was added and plotted as Log_{10} of % survival. Full and empty symbols represent untreated and treated cells respectively. Values are expressed as means of two biological replicates. Source data for Supplementary Figure 1 are provided as a Source Data file.

Protein	Name used in text	Construct	Experiments	Figures
ViaA	ViaA	AviTag-ViaA-His	SAXS, MALLS<br>BLI<br>Cellular EM<br>Dot-blots, TLC, GC-FID/MS<br>SDS-PAGE<br>Planar lipid bilayer	Figure 1<br>Figure 2<br>Not Shown<br>Figure 4, S3, S6<br>Figure S5<br>Figure S7
	His-ViaA	His-ViaA	Cellular EM, Wide-field	Figure 3,<br>Figure S2
	His-ViaA-AviTag	His-ViaA-AviTag	BLI	Figure 2
	NTV	His-NTV-AviTag	BLI	Figure S1
	PAmCherry-ViaA	His-PAmCherry-ViaA	PALM<br>Complementation Assay	Figure 3<br>Figure S8
	ViaA-PAmCherry	His-ViaA-PAmCherry	PALM<br>Complementation Assay	Figure 3<br>Figure S8
	PAmCherry-CTV	His-PAmCherry-CTV	PALM	Figure 3
	NTV-PAmCherry	NTV-PAmCherry-His	STORM	Figure S2
	$ViaA_{1-472}$	AviTag-$ViaA_{1-472}$-His	Dot-blots, TLC, GC-FID/MS<br>Cellular EM<br>SDS-PAGE	Figure 4, S3, S6<br>Figure S4<br>Figure S5
	$ViaA_{R476E/479E}$	AviTag-$ViaA_{R476E/479E}$-His	Dot-blots, TLC, GC-FID/MS<br>Cellular EM<br>SDS-PAGE	Figure 4, S3, S6<br>Figure S4<br>Figure S5
RavA			Cellular EM<br>Dot-blots, TLC, GC-FID/MS<br>SDS-PAGE<br>Planar lipid bilayer	Figure 3<br>Figure 4, S3, S6<br>Figure S5<br>Figure S7
	$RavA_{\Delta LARA}$	His- $RavA_{\Delta LARA}$	BLI<br>Dot-blots, TLC, GC-FID/MS<br>Cellular EM<br>SDS-PAGE	Figure S1<br>Figure 4, S3, S6<br>Figure S4<br>Figure S5
	BC2-RavA	His-BC2-RavA	STORM	Figure 3
	RavA-BC2	RavA-BC2-His	STORM	Figure 3
LdcI	LdcI	LdcI-His	Dot-blots	Figure S3

Supplementary Table 1. Summary of constructs used for experiments presented in each of the main and supplementary figures and their annotation in the main text.

Plasmid name	Description	Reference
pKD46	repA101(ts), AmpR, arabinose-inducible promoter (ParaB, araC) for expression of λ-Red casette genes (exo, gam, beta).	Datsenko & Wanner (2000), PNAS.
pKD3	AmpR, FRT-cat-FRT.	Datsenko & Wanner (2000), PNAS.
pCP20	repA101(ts), AmpR, FRT-cat-FRT, FLP recombinase overexpression from *S. cerevisiae*.	Cherepanov & Wackernagel (1995), Gene.
pET-22b	AmpR, Expression vector containing N-terminal pelB signal sequence and C-terminal His-Tag sequence, T7 promoter.	Novagen
p11	AmpR, Cloning vector derived from pET15b(+), Bacterial vector for expression of N-terminal 6xHis-TEV tagged proteins.	Generous gift by Prof. Walid Houry
pBAD-His-PAmCherry	Expression vector that contains genes encoding for PAmCherry protein and arabinose-inducible gene expression (ParaB, araC).	Subach *et al.*, (2009), Nature Methods.
pmCherry-N1	Expression vector that contains genes encoding for mCherry protein.	Clontech

Supplementary Table 2. List of plasmids used in this study.

Primer sequence	Template plasmid	Cloning method	Construct/Plasmid name
ACATATGCTAACGCTGGATACGCTTAATGT	*Genomic DNA: E.coli K12 - MG1655*	Ligation restriction Nde1/BamH1	His-ViaA
GGATCCTTATCGCCGCCAGCGTCTG			
	p11 backbone		
TACATATGGCTCACCCTCATTTATTAGCG	*Genomic DNA: E.coli K12 - MG1655*	Ligation restriction Nde1/BamH1	His-RavA
ATGGATCCTTAGCATTGTTGTGCCTGGCG			
	p11 backbone		
ATGCGCATATGCTAACGCTGGATACG	His-ViaA	Ligation restriction Nde1/BamH1	His-ViaA-AviTag
GCATAGGATCCTTATTCATGCCATTCAATTTTCTGCGCTTCAAAAATATCGTTCAGGCCTCGCCGCCAGCGTCTGAG			
	His-RavA		
CATCACAGCAGCGGCGTGAGCAAGGGCGAGGAG	pBAD His PamCherry	Gibson assembly	His-PAmCherry-ViaA
GTTTTCTCTGCCGCTCTTGTACAGCTCGTCCATGCC			
AGCGGCAGAGAAAACTTGTATTTCCAGGGTGGTGGCCATATGCTAACGCT	p11-His-ViaA		
TCCTTATCGCCGCCATAGGATCCTTATCGCCGCCAG			
TGGCGGCGATAAGGATCC	p11-His-ViaA (backbone)		
GCCGCTGCTGTGATGATG			
TTTTTGAAGCGCAGAAAATTGAATGGCATGAAGGCAGCGAAAACTTGTATTTCCAGGG	His-PAmCherry-ViaA	Gibson assembly	AviTag-ViaA-His
GTGCTCGAGTGCGGCCGCTCGCCGCCAGCGTCTGAG			
GCGGCCGCACTCGAGCAC	pET-22b		
AATTTTCTGCGCTTCAAAAATATCGTTCAGGCCCATATGTATATCTCCTTCTTAAAGTTAAAC			
TTTTTGAAGCGCAGAAAATTGAATGGCATGAAGGCAGCGAAAACTTGTATTTCCAGGG	His-PAmCherry-ViaA	Gibson assembly	AviTag-ViaA$_{1-472}$-His
GTGCTCGAGTGCGGCCGCCCCGGTATCAAAGCGCC			
GCGGCCGCACTCGAGCAC	pET-22b		
AATTTTCTGCGCTTCAAAAATATCGTTCAGGCCCATATGTATATCTCCTTCTTAAAGTTAAAC			
TGCTAACGCTGGATACGCTTAATG	His-PAmCherry-ViaA	Ligation restriction EcoR1/Not1	AviTag-ViaA$_{R476E/R479E}$-His
TTTGCGGCCGCTCGCCGCCAGCGTTCGAGCAGTTCGCTTCGCATCCCGGTATC			
	AviTag-ViaA-His		
TGCTAACGCTGGATACGCTTAATG	His-PAmCherry-ViaA		

TTTGCGGCCGCTCGCCGCCAGCGGGCGAGCAGGGCGCTTCGCATCCCGGTATC		Ligation restriction EcoR1/Not1	AviTag-ViaA$_{R476A/R479A}$-His
	AviTag-ViaA-His		
TGCCAATGGCTCACGGCCGCTTTACGATCCGGGCCGCTGCTGTGATGATG	His-RavA-EC	Gibson assembly	His-BC2-RavA
GATACCTACAGCGTGAGCTATGAGAAAG			
CGTGAGCCATTGGCAGCAGGAAAACTTGTATTTCCAGGGCC	His-RavA-EC		
CTCACGCTGTAGGTATCTCAGTTCGGTG			
CAGGCACAACAATGCGGCTCAGGCGGTGGTTCAG	LdcI-BC2-His-pET22b	Gibson assembly	RavA-BC2-His
CATATGTATATCTCCTTCTTAAAGTTAAACAAAATTATTTCTAGAGG			
GCATTGTTGTGCCTGGCGAA	His-RavA-Dendra2T69A-p11		
CTTTAAGAAGGAGATATACATATGGCTCACCCTCATTTATTAGCGGAA			
CATATGTATATCTCCTTCTTAAAGTTAAACAAAATTATTTCTAGAGG	pET-22b	Gibson assembly	NTV-PAmCherry-His
AATTCGAGCTCCGTCGACAAG			
AAGAAGGAGATATACATATGCTAACGCTGGATACGCTTAATGTGATGC	His-ViaA-PAmCherry		
AAATACAAGTTTTCACCGCTATGCACCACCGGGCGTTC			
AGCGGTGAAAACTTGTATTTCCAGGGCGGTGTGAGCAAGGGCGAGGAG	His-ViaA-PAmCherry		
TTGTCGACGGAGCTCGAATTCTTGTACAGCTCGTCCATGC			
TGTATTTCCAGGGTGGTGGCAAAGATTACGACGAACAGCCGC	His-PAmCherry-ViaA	Gibson assembly	His-PAmCherry-CTD
CTCACGCTGTAGGTATCTCAGTTCGGTG			
GATACCTACAGCGTGAGCTATGAGAAAG	His-PAmCherry-ViaA		
GCCACCACCCTGGAAATACA			
TGGCGGCGATAAGGATCCTAATAAC	p11-His-ViaA	Gibson assembly	His-ViaA-PAmCherry
ACCGCCCTGGAAATACAAGTTTTCACCGCTTCGCCGCCAGCGTCTGAG			
AGCGGTGAAAACTTGTATTTCCAGGGCGGTGTGAGCAAGGGCGAGGAG	pBAD His PamCherry		
TCCTTATCGCCGCCATTACTTGTACAGCTCGTCCATGCC			

ACGTCTTGAGCGATTGATGCTAACGCTGGATACGCT	AviTag-ViaA$_{1-472}$-His	Gibson assembly	pKD3_ViaA$_{1-472}$
AGCTCCAGCCTACACTCACCCGGTATCAAAGCGCCAG			
CAATCGCTCAAGACGTGTAATG	pKD3		
GTGTAGGCTGGAGCTGCTTC			

GCGGCCGCACTCGAGCAC	AviTag-ViaA$_{R476E/R479E}$	Gibson assembly	pKD3_ViaA$_{R476E/R479E}$
AGCTCCAGCCTACACTCACCCGGTATCAAAGCGCCAG			
CAATCGCTCAAGACGTGTAATG	pKD3		
GTGTAGGCTGGAGCTGCTTC			
ACGTCTTGAGCGATTGATGGCTCACCCTCATTTATTAGC	His-RavA$_{\Delta LARA}$_p11 plasmid *(from Walid 2017)*	Gibson assembly	pKD3_RavA$_{\Delta LARA}$
AGCTCCAGCCTACACTTAGCATTGTTGTGCCTGGC			
CAATCGCTCAAGACGTGTAATG	pKD3		
GTGTAGGCTGGAGCTGCTTC			

Primer sequence	Template plasmid	Cloning method	Target strain | Final mutant stain
GTATGGCCAGCTGCTGTTCGCGAGAGCGTCCCTTCTCTGCTGTAAGATGGTCCATATGAATATCC	pKD3_ViaA$_{1-472}$	Recombineering	MG1655-*ΔravA/ΔviaA* | MG1655-*ΔravA/viaA$_{1-472}$*::cat
CTCGCAATTTACGCAGAACTTTTGACGAAAGGACGCCACTTCATtATGCTAACGCTGGATACGCT			
GTATGGCCAGCTGCTGTTCGCGAGAGCGTCCCTTCTCTGCTGTAAGCCATGGTCCATATGAATATCC	pKD3_ViaA$_{R476E/R479E}$	Recombineering	MG1655-*ΔravA/ΔviaA* | MG1655-*ΔravA/viaA$_{R476E/R479E}$*::cat
CTCGCAATTTACGCAGAACTTTTGACGAAAGGACGCCACTTCATtATGCTAACGCTGGATACGCT			
GTATGGCCAGCTGCTGTTCGCGAGAGCGTCCCTTCTCTGCTGTAAGCCATGGTCCATATGAATATCC	pKD3_RavA-ΔLARA	Recombineering	MG1655-*ΔravA/ΔviaA* | MG1655-*ravAΔLARA/ΔViaA*::cat
CTCGCAATTTACGCAGAACTTTTGACGAAAGGACGCCACTTCATtATGGCTCACCCTCATTTATTAGC			

Supplementary Table 3. Summary of primers, templates and cloning strategies to obtain the various constructs, plasmids and mutant strains used in this study.
