# Supplementary material for: The AAA+ ATPase RavA and its binding partner ViaA modulate E. coli aminoglycoside sensitivity through interaction with the inner membrane
Source: Nat Commun. 2022 Sep 20;13:5502. doi: 10.1038/s41467-022-32992-9 (PMC9489729; doi:10.1038/s41467-022-32992-9)
Supplement: Supplementary file 3 — Reporting Summary [file 41467_2022_32992_MOESM3_ESM.pdf]

nature portfolio

Corresponding author(s): Irina Gutsche

Last updated by author(s): Jul 14, 2022

Reporting Summary

Nature Portfolio wishes to improve the reproducibility of the work that we publish. This form provides structure for consistency and transparency in reporting. For further information on Nature Portfolio policies, see our Editorial Policies and the Editorial Policy Checklist.

Statistics

For all statistical analyses, confirm that the following items are present in the figure legend, table legend, main text, or Methods section.

n/a	Confirmed	
☐	☒	The exact sample size (*n*) for each experimental group/condition, given as a discrete number and unit of measurement
☐	☒	A statement on whether measurements were taken from distinct samples or whether the same sample was measured repeatedly
☐	☒	The statistical test(s) used AND whether they are one- or two-sided<br>*Only common tests should be described solely by name; describe more complex techniques in the Methods section.*
☒	☐	A description of all covariates tested
☒	☐	A description of any assumptions or corrections, such as tests of normality and adjustment for multiple comparisons
☐	☒	A full description of the statistical parameters including central tendency (e.g. means) or other basic estimates (e.g. regression coefficient) AND variation (e.g. standard deviation) or associated estimates of uncertainty (e.g. confidence intervals)
☐	☒	For null hypothesis testing, the test statistic (e.g. *F*, *t*, *r*) with confidence intervals, effect sizes, degrees of freedom and *P* value noted<br>*Give P values as exact values whenever suitable.*
☒	☐	For Bayesian analysis, information on the choice of priors and Markov chain Monte Carlo settings
☒	☐	For hierarchical and complex designs, identification of the appropriate level for tests and full reporting of outcomes
☒	☐	Estimates of effect sizes (e.g. Cohen's *d*, Pearson's *r*), indicating how they were calculated

Our web collection on statistics for biologists contains articles on many of the points above.

Software and code

Policy information about availability of computer code

Data collection	MALLS: ASTRA 6.1, BLI: ForteBio Data Aquisition 9.0, STORM/PALM Imaging: Metamorph 7.1, Wide Field Imaging: Volocity 7, TEM: Digital Micrograph (GATAN).
Data analysis	MALLS: ASTRA6.1, SEDFIT, SAXS: ATSAS 3.0, ScÅtter 4.0, SAXSMoW 2, BLI: FortéBio Data Analysis 9.0 , Densitometric analysis of SDS-PAGE: Image Lab 6.1, STORM/PALM Imaging: Thunderstorm 1.3 (ImageJ), Planar lipid bilayer experiments: QuB.

For manuscripts utilizing custom algorithms or software that are central to the research but not yet described in published literature, software must be made available to editors and reviewers. We strongly encourage code deposition in a community repository (e.g. GitHub). See the Nature Portfolio guidelines for submitting code & software for further information.

Data

Policy information about availability of data

All manuscripts must include a data availability statement. This statement should provide the following information, where applicable:

- Accession codes, unique identifiers, or web links for publicly available datasets
- A description of any restrictions on data availability
- For clinical datasets or third party data, please ensure that the statement adheres to our policy

All data presented in this study are included in the published article, supplementary information and source data file, and are available from the corresponding author on request.

Human research participants

Policy information about studies involving human research participants and Sex and Gender in Research.

Reporting on sex and gender	N/A
Population characteristics	N/A
Recruitment	N/A
Ethics oversight	N/A

Note that full information on the approval of the study protocol must also be provided in the manuscript.

Field-specific reporting

Please select the one below that is the best fit for your research. If you are not sure, read the appropriate sections before making your selection.

☒ Life sciences ☐ Behavioural & social sciences ☐ Ecological, evolutionary & environmental sciences

For a reference copy of the document with all sections, see nature.com/documents/nr-reporting-summary-flat.pdf

Life sciences study design

All studies must disclose on these points even when the disclosure is negative.

Sample size	Due to time and experimental constraints, we chose to perform at least 3 biological replicates for all but one experiment performed in this study.
Data exclusions	No data were excluded from the analyses.
Replication	All BLI binding studies were performed in triplicate (technical triplicates). Lipid quantification analysis was done in 5 biological replicates for the WT proteins, 7 for ViaA-OE and 3 for the other constructs. All attempts to replicate were successful. No additional experiments were performed other than the ones described in this study
Randomization	Due to the low number of replicates, randomization is not applicable here.
Blinding	Due to the low number of replicates and to avoid mislabeling, no samples were blinded.

Reporting for specific materials, systems and methods

We require information from authors about some types of materials, experimental systems and methods used in many studies. Here, indicate whether each material, system or method listed is relevant to your study. If you are not sure if a list item applies to your research, read the appropriate section before selecting a response.

Materials & experimental systems

n/a	Involved in the study	
☐	☒	Antibodies
☒	☐	Eukaryotic cell lines
☒	☐	Palaeontology and archaeology
☒	☐	Animals and other organisms
☒	☐	Clinical data
☒	☐	Dual use research of concern

Methods

n/a	Involved in the study	
☒	☐	ChIP-seq
☒	☐	Flow cytometry
☒	☐	MRI-based neuroimaging

Antibodies

Antibodies used	Anti-ViaA rat polyclonal antibody (Qalam-Antibody), anti-RavA rabbit polyclonal antibody (Qalam-Antibody), anti-LdcI rabbit polyclonal antibody (Qalam-Antibody), anti-rabbit IgG secondary antibody conjugated with horseradish peroxidase (Merck), anti-rat IgG secondary antibody conjugated with horseradish peroxidase (Merck).
Validation	All antibodies used in this study were validated for use in Western Blotting.
