# Supplementary figures and images for: The AAA+ ATPase RavA and its binding partner ViaA modulate E. coli aminoglycoside sensitivity through interaction with the inner membrane
Source: Nat Commun. 2022 Sep 20;13:5502. doi: 10.1038/s41467-022-32992-9 (PMC9489729; doi:10.1038/s41467-022-32992-9)

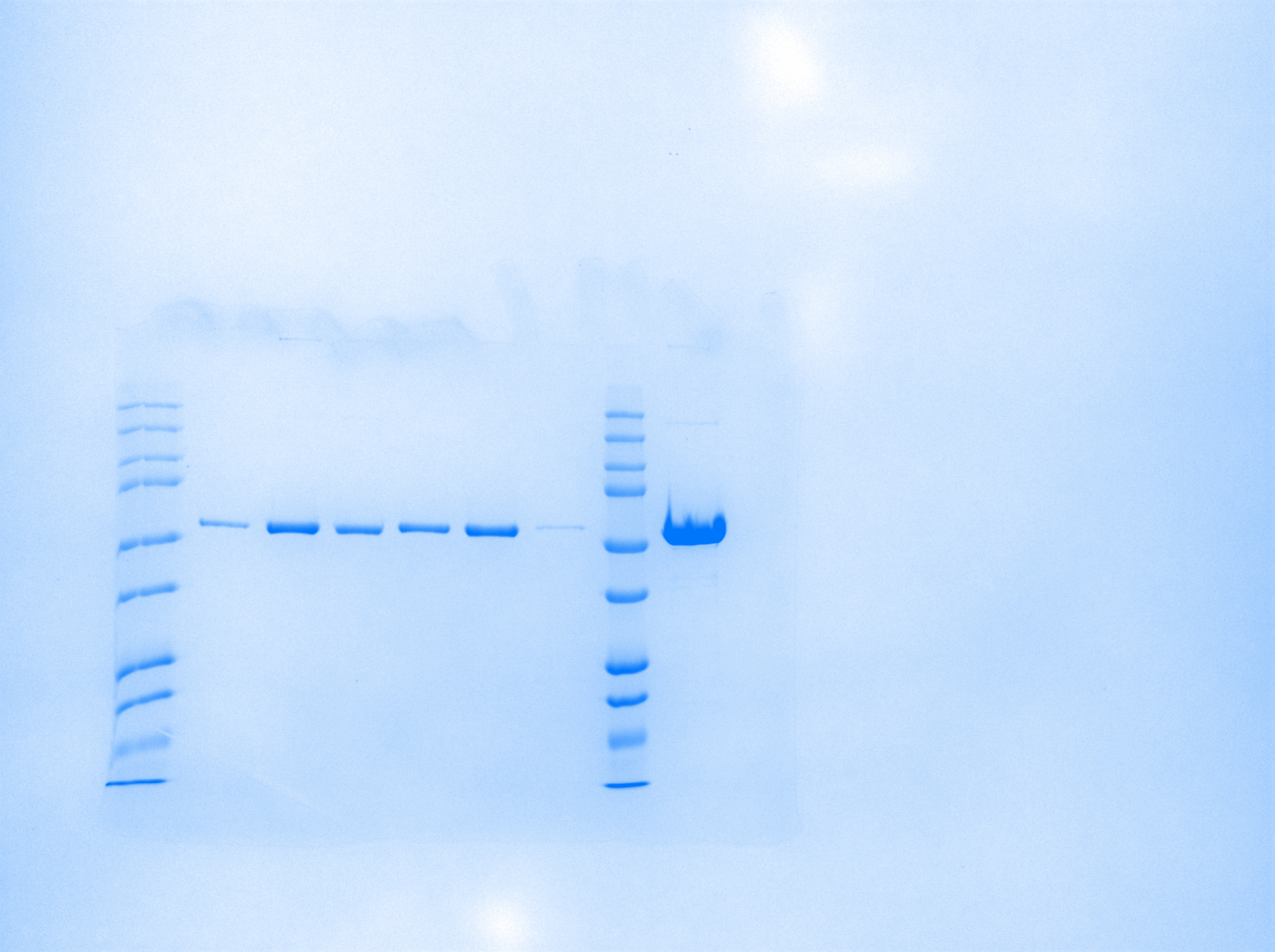

Supplement: Supplementary file 4 — Source Data [file 41467_2022_32992_MOESM4_ESM.zip › Source_Data/Figure1/SDSPAGE/Figure1B.tif]

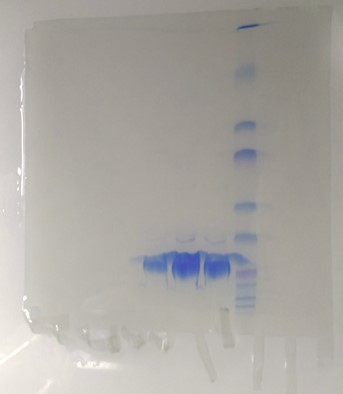

Supplement: Supplementary file 4 — Source Data [file 41467_2022_32992_MOESM4_ESM.zip › Source_Data/Figure2/SDSPAGE/Figure2C_FrdA.jpg]

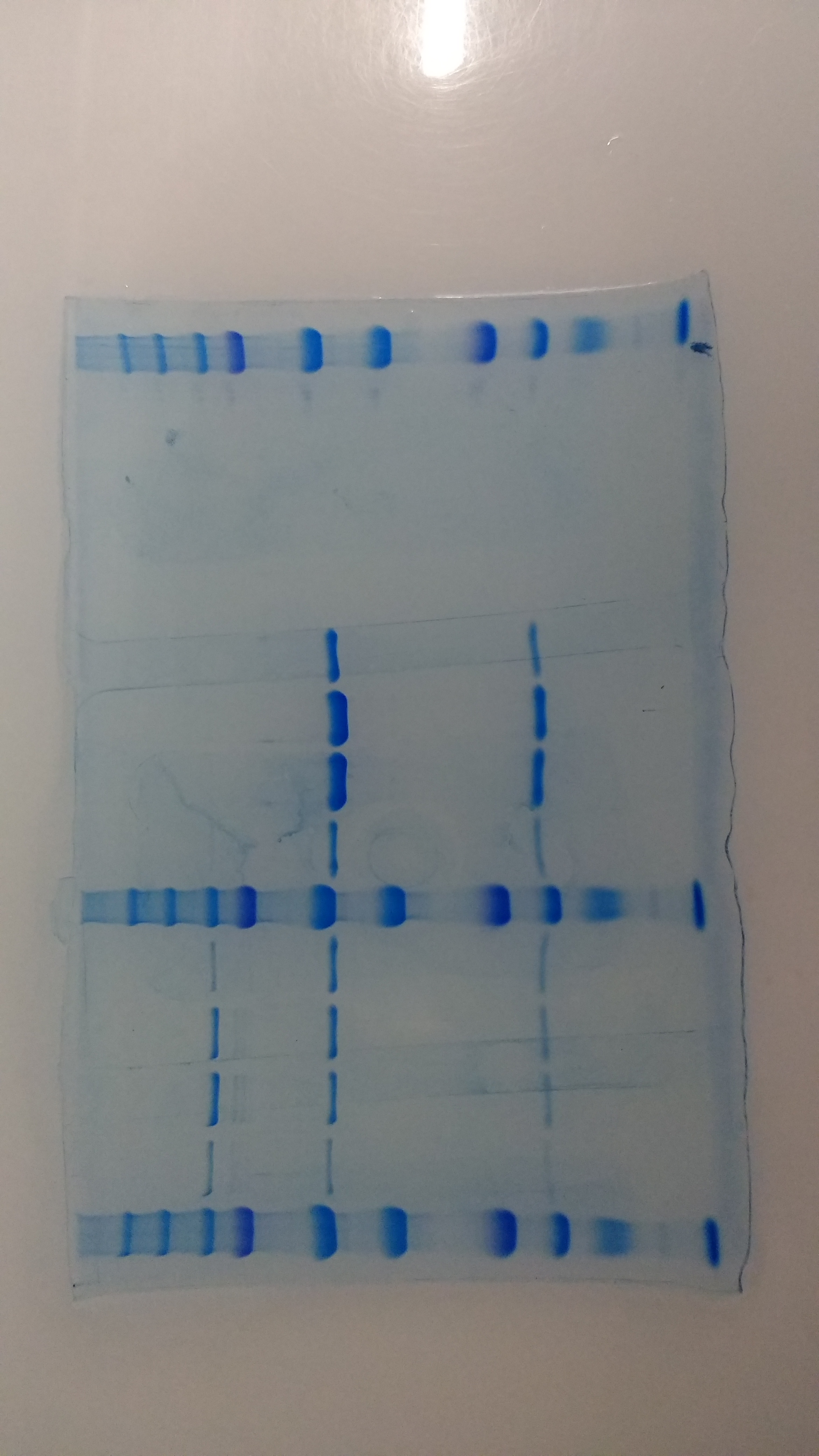

Supplement: Supplementary file 4 — Source Data [file 41467_2022_32992_MOESM4_ESM.zip › Source_Data/Figure2/SDSPAGE/Figure2C_NuoEFG_NuoEF.jpg]
